# *En route* to personalized medicine: uncovering distinct IgE reactivity pattern to house dust mite components in Brazilian and Austrian allergic patients

**DOI:** 10.1002/clt2.12004

**Published:** 2021-03-24

**Authors:** Carina S. Pinheiro, Eduardo S. Silva, Emília M. M. de Andrade Belitardo, Luis G. C. Pacheco, Eric R. G. R. Aguiar, Neuza M. Alcantara‐Neves, Gabriele Gadermaier, Fatima Ferreira

**Affiliations:** ^1^ Institute of Health Sciences – ICS Federal University of Bahia Salvador Brazil; ^2^ Department of Biological Sciences Santa Cruz State University Ilhéus Brazil; ^3^ Department of Biosciences University of Salzburg Salzburg Austria

**Keywords:** allergens, allergy, mites, molecular diagnosis

## Abstract

**Aim:**

Molecular sensitization profile analyses of allergic individuals to the house dust mites (HDM) *Blomia tropicalis* and *Dermatophagoides pteronyssinus* from Brazil and Austria, in the attempt to comprehend the individual contribution of the molecular components in the diagnosis of HDM allergy.

**Methodology:**

These analyses were made using a new in vitro multiplex allergen assay which allows simultaneous measurement of specific IgE against the whole allergen extract as well its components.

**Results and Conclusion:**

The data showed that in Brazil the inclusion of the molecular components Blo t 5 and/or Blo t 21 major allergens and Blo t 2 can increase the sensitivity and specificity of the assay for the diagnosis of allergy to *B. tropicalis*, using matrix‐based methodologies. Also we highlighted, for the first time, the importance of Blo t 2 analysis for a sensitive diagnosis, since some individuals were sensitized only to this molecular component. Regarding the sensitization profile of individuals sensitized to *D. pteronyssinus*, we point out the importance of analyzing the molecular components Der p23 and Der p 7, in addition to Der p 1 and Der p 2 for an accurate diagnosis based on matrices.

To the Editor,

The prevalence of allergic diseases has significantly increased worldwide in the last few decades. The most common form of allergy in tropical countries is diagnosed by skin prick test to detect skin sensitization and/or quantification of serum IgE against house dust mites allergens (HDM).^1^ However, these methods can show variability due to the different sources of allergens and preparations resulting in different extract compositions.^2^ To overcome this problem, an alternative for a correct diagnosis of allergies is the detection of IgE against a panel of allergenic molecules.^3^, ^4^ In this context, the present study aims to analyze the molecular sensitization profile of allergic individuals to the HDM *Blomia tropicalis* and *Dermatophagoides pteronyssinus* in an attempt to comprehend the individual contribution of the molecular components in the diagnosis of HDM allergy in different populations.

The sensitization profile was obtained from HDM allergic individuals from two countries; from Salvador city, Northeast Brazil (*n* = 44) sensitized to *B. tropicalis* and *D. pteronyssinus;* and from Vienna, Austria (*n* = 23) sensitized to *D. pteronyssinus*. The analyses were made using a new in vitro multiplex allergen assay customized called ALEX® (MadX macroarray diagnostic) containing six allergens from *B. tropicalis,* (rBlo t 1, 2, 5, 21 and two different Blo t Extract) and eight from *D. pteronyssinus,* (Der p 1, 2, 5, 10, 11, 23, and Der p Extract). From *B. tropicalis* the recombinant allergens and one extract were provided by our group. sIgE levels were measured in individuals of both countries and the results were divided in the sensitization profile of mite allergic individuals to *B. tropicalis* and *D. pteronyssinus* (Figure 1A). Through the analyses of the sIgE levels to *B. tropicalis* and *D. pteronyssinus*, it is possible to observe that the use of specific molecular components can be used to identify individuals of a specific country. Blo t 5 and 21 reactivity can be used to clustering Brazilian individuals, where the highest sensitivity among the *B. tropicalis* components included in the panel was found for Blo t 21 with 74.47%, followed by Blo t 5 (Figure 1B). Furthermore, Der p 1 and 2 reactivity can be used to clustering Austrian individuals (Figure 1B). The analysis of the molecular components sensitization in all individuals from Austria and Brazil from group 1 (Der p 1and Blo t 1), shows that the group 1 allergens have a partial cross‐reactivity, and Der p 1 is predominant over Blo t 1. In‐group 2 allergens there is higher cross‐reactivity, but it is also possible to regard exclusive sensitization to Der p 2 and Blo t 2 (Figure 1C). All individuals with sIgE (kUA/L) > 0.19 in ALEX® where considered positive. Therefore, from 44 sera of Brazilian individuals, 42 and 35 were positive to total extract or components of *B. tropicalis* and *D. pteronyssinus* respectively, and from 23 sera of Austrian individuals, 21 and 23 individuals were positive, to total extract or components of *B. tropicalis* and *D. pteronyssinus* respectively. These results highlighted the presence of cross‐reaction, due to the number of positive individuals to *B. tropicalis* detected in Austria, a country without register of *B. tropicalis* mite.

**FIGURE 1:**
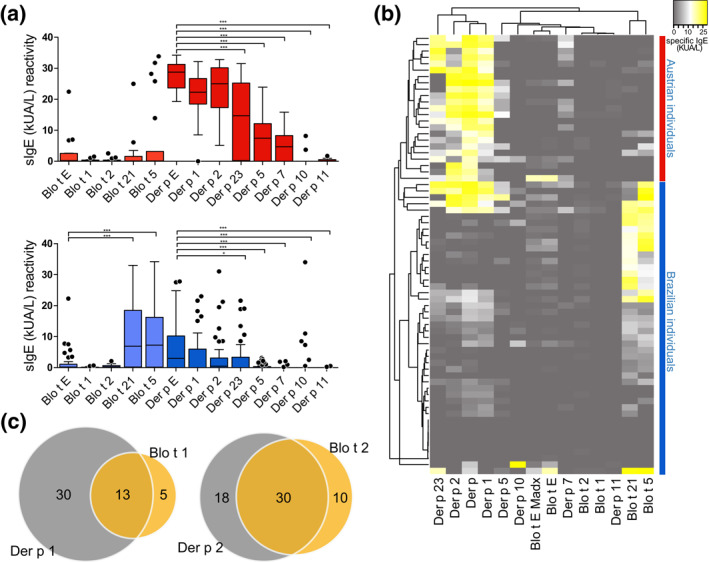
Sensitization profile of Austrian (Red) and Brazilian (Blue) individuals with HDM allergy. (A) Levels of sIgE to HDM components and extract, differences are represented as *p* < 0.001 (***), and *p* < 0.05 (*). (B) A two‐way hierarchical clustering analysis heat map based on sIgE levels of 14 allergens. (C) The total number of patients positive in Austria and Brazil to group 1 are 48 individuals and to group 2 are 58 individuals and they are distributed as show in the Venn diagram. Letter represents the total extract (E) and numbers represent the allergen classes. HDM, house dust mites

The individual contribution of each component and the extract was visualized in a graphic of cumulative frequency, revealing that to achieve 50% of positive individuals to *B. tropicalis* and *D*. *pteronyssinus* in Brazilian individuals the combination of molecular components analyzed should be different and higher than in Austrian (Figure 2A). In addition, it was possible to observe a statistically significant positive correlation (*p* < 0.0001) between IgE level to Blo t Extract and Blo t 5 (*r* = 0.60) and Blo t 21 (*r* = 0.59) (Figure 2B). The importance of the Blo t 5 and Blo t 21 was highlighted by our group in a previously article.^5^ However in endemic areas to *B. tropicalis,* the inclusion of the molecular components Blo t 2 are also necessary to achieve a more sensitive diagnosis, since some individuals are sensitized exclusively by this molecular component (Figure 2A). Regarding *D. pteronyssinus* sensitization Der p Extract and the molecular components Der p 1 and Der p 2 were shown to be able to detect more than 95% of the positive individuals in Austria, and showed higher levels of IgE levels when compared with the other analyzed molecular components (Figure 1A). In Brazilian allergic individuals, Der p Extract was able to detect 78% of the positive individuals and Der p 2 71%. Besides, it was also possible to observe a positive correlation between IgE levels for Der p Extract with Der p 1 (*r* = 0.80 and *r* = 0.83) and Der p 2 (*r* = 0.53 and *r* = 0.79) for Austrian and Brazilian individuals respectively (Figure 2B). These results are in accordance with the literature showing the importance of these two molecular components in the IgE sensitization.^6^ However, concerning Brazilian individuals other molecular components should also be analyzed for a complete diagnosis, such as Der p 7, and Der p 23.^7^ The importance of Der p 23 as one of the main allergens in the IgE sensitization has already been pointed out in several articles, however it is the first time that we have shown the importance of this molecular component in Brazilian individuals. Where Der p 23 presented a positive correlation to specific IgE level production anti‐Der p Extract (*r* = 0.75, *p* < 0.0001 Figure 2B).

**FIGURE 2:**
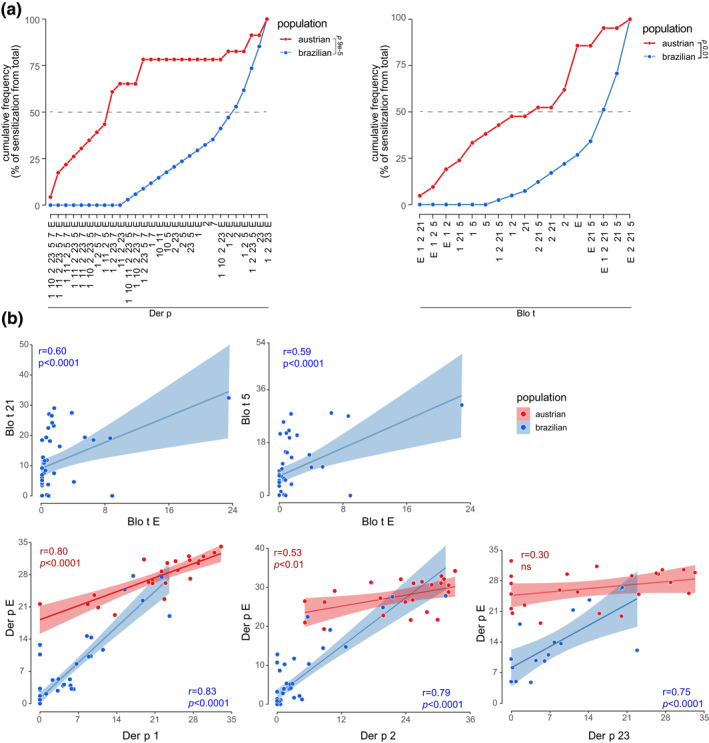
Contribution of the *Blomia tropicalis* and *Dermatophagoides pteronyssinus* molecular components and total extracts to sIgE sensitization. Austrian individuals (Red) and Brazilian individuals (Blue). (A) Cumulative frequency of sensitization with the molecular components and total extract of *D. pteronyssinus* and *B. tropicalis*. Numbers represent the allergen classes Der p (1, 2, 5, 7, 10, 11, and 23) and Blo t (1, 2, 5, and 21); letter represents the total extract (E). (B) Scatter plot associated to correlation analysis assessing relationship between serum sIgE levels of house dust mite extracts and their molecular components

In conclusion, our study emphasized the importance of using the components Blo t 5, Blo t 21 and Blo t 2 of *B. tropicalis* to increase the sensitivity and specificity of the assay to diagnosis allergy to this acarid, based on arrays, once was observed a cross‐reaction between the two mites.^8^ Furthermore, our observations also highlighted the importance of the analysis of the molecular components Der p 23 and Der p 7, besides Der p 1 and Der p 2, since in some individuals it alone and not the extract was responsible for the sensitization. However, even with all the advantages related to the identification of the simultaneous molecular components and extract, it is important to continuously improve the array methodology, since ImmunoCAP remains the gold standard.

## AUTHOR CONTRIBUTIONS

Carina S. Pinheiro carried out experiments and participated in the analysis of the results. Eduardo S. Silva and Emilia M. M. de Andrade Belitardo helped with Brazilian serum analyzes. Luis G. C. Pacheco, Eric G. R. Aguiar and Neuza M. Alcantara‐Neves participated in results analyzes and graphic design. Carina S. Pinheiro, Gabriele Gadermaier and Fatima Ferreira conceived the study, participated in its design and coordination, and helped to draft the manuscript. All authors read and approved the final manuscript.
